# Direct and indirect effects of predation and parasitism on the *Anopheles gambiae* mosquito

**DOI:** 10.1186/s13071-020-3915-8

**Published:** 2020-01-30

**Authors:** Fedinand Ong’wen, Patrick Ogola Onyango, Tullu Bukhari

**Affiliations:** Department of Zoology, School of Physical and Biological Sciences, Maseno University, Maseno, Kenya

**Keywords:** Predator, Parasite, *Anopheles*, Susceptibility, Malaria

## Abstract

**Background:**

A good understanding of mosquito ecology is imperative for integrated vector control of malaria. In breeding sites, *Anopheles* larvae are concurrently exposed to predators and parasites. However, to our knowledge, there is no study on combined effects of predators and parasites on development and survival of larvae and their carry-over effects on adult survivorship and susceptibility to further parasite infection.

**Methods:**

This study focused on effects of the nymphs of the dragonfly *Pantala flavescens* and the parasitic fungus *Beauveria bassiana* on *Anopheles gambiae*, to determine: predation efficacy of nymphs against *An. gambiae* larvae; development rate of *An. gambiae* larvae in the presence of one, two or four constrained nymphs; efficacy of *B. bassiana* against *An. gambiae* larvae at doses of 3, 6 and 12 mg; and survival of adult mosquitoes exposed to *B. bassiana*, following pre-exposure to a constrained predator and/or parasite at the larval stage. The experiments consisted of survival bioassays quantified as pupation day, or dead larvae and/or adults.

**Results:**

Nymphs had an average predation efficacy of 88.3% (95% CI: 87.5–89.1) at 24 hours, against *An. gambiae* larvae. The presence of one or two nymphs reduced development time of larvae by 0.65 and 0.35 days, respectively. However, development time of larvae exposed to four nymphs was similar to the control larvae. Larvae exposed to 3, 6 and 12 mg of *B. bassiana* were 2.0, 2.5 and 3.5 times more likely to die, respectively, compared to control larvae. Adults not pre-exposed, those pre-exposed to predator, parasite, or both were 45.8, 67.4, 50.9 and 112.0 times more likely to die, respectively, compared to control that were unexposed to predator or parasite, at larval and adult stage.

**Conclusions:**

This study shows that both predator and parasite can reduce larval population of *An. gambiae*, and presence of predator cues decreases development time in breeding sites, as well as, increases the susceptibility of emerging adult to fungus. Predator and parasite both have an additive effect on survival of adults exposed to *B. bassiana*. Field studies are required for an in-depth understanding of predator and parasite influence on mosquito development time, survival and susceptibility in nature.
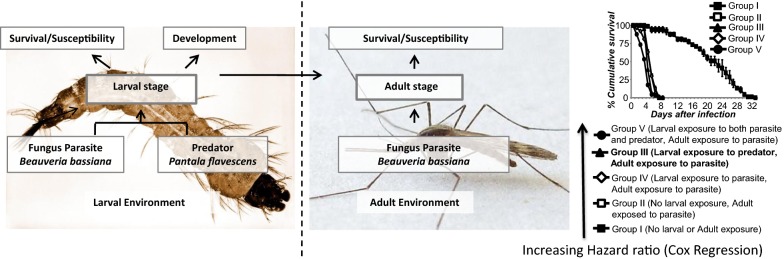

## Background

In 2017, the World Health Organization (WHO) estimated 219 million cases and 435,000 deaths due to malaria. This is 20 million less than the number of malaria cases in 2010. However, data show that from 2015–2017 there has been no significant reduction in global malaria cases [1]. Human malaria is caused by *Plasmodium* spp. transmitted by *Anopheles* mosquitoes. Current malaria mosquito control strategies mainly rely on insecticides and target female *Anopheles* mosquitoes indoors. Outdoor malaria transmission, insecticide resistance and non-target effects challenge current mosquito control programmes [2]. There is, therefore, an increased interest in novel mosquito control strategies and a general understanding that integrated vector management (IVM) is required for a sustainable and environmentally friendly mosquito control programme [2, 3].

A good understanding of mosquito ecology is a precondition for IVM to eliminate and eradicate the malaria mosquito [4, 5]. *Anopheles* larvae are aquatic and are found in a variety of breeding sites in terms of size, permanence, vegetation and water cleanliness. Studies have shown that there is high larval mortality in the natural breeding sites due to climatic conditions, parasitism and predation [6–8]. This high mortality can exert a high selection pressure in natural habitats and modify how the emerging adults are equipped for future climate, parasite and predator challenges in their terrestrial environment. It is known that a high proportion of the *An. gambiae* female population is resistant to infection with *Plasmodium* parasites and therefore does not contribute to malaria transmission. This resistance is genetic and linked to the immune response of the mosquitoes [9]. Non-genetic factors, e.g. climate, predators and parasites can also influence the resistance of mosquitoes to parasites [10]. Predation (presence of predation reduces phenoloxidase activity) and climatic conditions (appropriate environmental temperature can favor the immune response) indirectly influence the immune system while parasites can directly or indirectly influence the immune system [11, 12]. These indirect non-lethal effects of both parasites and predators can influence the fecundity, survival and immune response of the surviving individuals and consequently malaria transmission [13–17]. A study conducted by Bargielowski and Koella [13], showed that infection at the larval stage with the microsporidian *Vavraia culicis* increased the resistance of adult *Anopheles* mosquitoes to *Plasmodium* infection. However, *An. stephensi* females exposed to the fungus *Beauveria bassiana* at the larval stage and re-exposed to the same fungus during the adult stage had equal survival as females exposed only during the adult stage showing no influence of larval exposure on adult susceptibility [18]. Another study showed that *An. gambiae* larvae exposed to the entomopathogenic fungus *Aspergillus parasiticus* developed into adults that had reduced longevity and fecundity [15]. Roux et al. [16] observed that exposure to the predator backswimmer *Anisop jaczewskii* did not influence the susceptibility of *An. coluzzii* to *Plasmosium falciparum* but increased the larval development time, reduced the female wing size as well as influenced the fecundity and longevity. Similarly, Chobua et al. [19] showed that development time of *An. gambiae* larvae increased in the presence of the predator fishes *Carassius auratus* and *Gambusia affinis*. Also, the presence of predator cues reduced oviposition and resulted in the emergence of more female mosquitoes compared to male [19].

To date, studies on this subject have not looked at the combined effect of predators and parasite presence during the larval stage [13, 15, 16]. In nature, however, the larvae can be concurrently exposed to both predators and parasites. This study reports the lethal and non-lethal effects of both predation and parasitism on the larval stage of *An. gambiae* and the carry-over effects of these factors on the susceptibility of the adult mosquitoes to a subsequent exposure to the same parasite. In this study we used the dragonfly *Pantala flavescens* and the entomopathogenic fungus *B. bassiana* as the predator and parasite of *An. gambiae*, respectively.

The dragonfly *P. flavescens* is widespread (except in Antarctica), although rare in Europe, and the nymph has been associated with low densities or absence of *Anopheles* larvae in otherwise suitable breeding sites [20]. Also, in the laboratory *P. flavescens* nymphs were found to be efficient predators with one nymph feeding on 54 ± 5 *Aedes aegypti* larvae within 24 hours [21]. Since 2003, entomopathogenic fungi have gained a renewed interest for their potential to control malaria mosquito larvae and adults [22–26]. Many laboratory and field studies have been conducted focusing on improving the delivery of fungal spores to the mosquitoes and the potential to control insecticide-resistant mosquitoes [22, 24, 27–29]. Multiple strains of *Metarhizium anisopliae* and *B. bassiana* have been intensively studied in this regard [18, 22–30].

The main objective of this study was to investigate the direct and indirect effects of the nymphs of the dragonfly *P. flavescens* and the parasitic fungus *B. bassiana* on the development and survival of *An. gambiae* larvae, as well as the susceptibility to the fungus of mosquito adults, that developed from the larvae exposed to *P. flavescens* and/or *B. bassiana*. The specific objectives were to determine: the predation efficacy of *P. flavescens* nymphs against *An. gambiae* larvae; the development rate of *An. gambiae* larvae reared in the presence of varying densities of constrained *P. flavescens* nymphs; the efficacy of *B. bassiana* against *An. gambiae* larvae at doses of 3, 6, and 12 mg; and the survival of adult mosquitoes that emerged from water with the predator *P. flavescens* nymph, with the parasite *B. bassiana*, or with both predator and parasite following a second exposure to *B. bassiana* at the adult stage.

## Methods

Mosquito rearing and experiments were performed at the Animal House Insectary, Department of Zoology in Maseno University, Kenya, from January 2017 to April 2017. During this period, room temperature ranged between 26–32 °C and relative humidity ranged between 65–70%.

### *Anopheles gambiae* rearing

*Anopheles gambiae* eggs were obtained from the Kenya Medical Research Institute, Kisian (courtesy of Dr Andrew Githeko). Eggs were added to 1 liter of dechlorinated tap water in plastic bowls (20 × 15 × 5 cm), lined with No. 1 Whatman filter paper to prevent eggs from adhering to the sides of the plastic bowls and drying out.

The hatched larvae were fed on Liquifry No. 1 (Interpet Ltd., Dorking, Surrey, UK) (1 g daily) for the first two days and then on ground cat food (Purina, Go cat®, UK) for the rest of the larval period [31]. The developing pupae were removed with a plastic pipette and placed in 300 ml clear plastic cups inside holding cages (30 × 30 × 30 cm) for the adults to emerge. All adults were fed on 6% glucose soaked in cotton wool.

### *Pantala flavescens* nymph rearing

*Pantala flavescens* dragonfly nymphs were captured from rice fields in the Ahero Irrigation Scheme, Ahero, Kenya and identified according to Paul and Kakkassery [32]. Two hundred nymphs were collected and transported to the Animal House Insectary, at Maseno University. Nymphs that weighed below 2.5 g each were kept together in bowls (20 × 15 × 5 cm) with 250 ml water. Five nymphs were kept in each bowl until they weighed 2.5 g or more. Nymphs were fed on 100 third-instar larvae in the larval bowls (the average daily number of larvae (20 larvae) fed on by each nymph below 2.5 g had been pre-determined earlier). Each nymph, that weighed 2.5 g or above, was kept, separately, in a 300 ml clear plastic cup containing 50 ml dechlorinated tap water. The nymphs were fed on 13–14 third-instar mosquito larvae per day, which is half their daily larval consumption, aimed at keeping them alive and active in predation. The uneaten larvae were removed from the cups or bowls once they pupated. Any nymph used in an experiment had to weigh 3 g ± 1 mg. Each nymph was starved for 24 h prior to each experiment.

### *Beauveria bassiana* spores

Spores of *B. bassiana* (Strain GHA Technical Active Ingredient, CAS 63428-82-0, Lam International Corporation, Butte, USA) were stored in an airtight plastic container at 4 °C. The germination percentage of spores was tested prior to the experiments and only the spores with > 85% germination were used [28].

### Predation efficacy of *P. flavescens* nymph against *An. gambiae* larvae

This experiment had one control group and one treatment group. Each group was replicated four times. Each replicate consisted of 30 third-instar larvae placed in a larval bowl with 1 liter of dechlorinated tap water. In the control replicates, larvae were not exposed to nymphs of *P. flavescens*. In each treatment replicate, larvae were exposed to one *P. flavescens* nymph. In all the groups, the number of surviving larvae was recorded after every hour for the first 12 h, and then, after 24 h.

### Development rate of *An. gambiae* larvae reared in the presence of varying densities of constrained *P. flavescens* nymphs

**Fig. 1:**
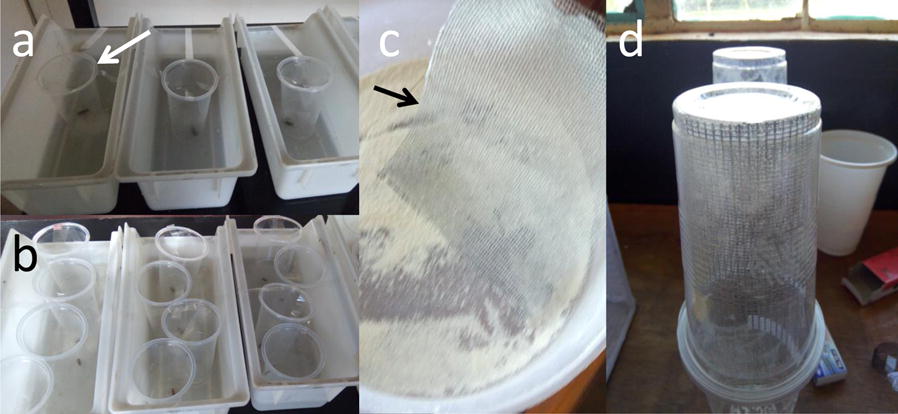
Bioassay set-up. **a** Exposure of *Anopheles gambiae* larvae to one constrained *Pantala flavescens* in plastic cups (arrow) inside the larval bowl. **b** Exposure of *An. gambiae* larvae to four constrained *P. flavescens* kept in separate plastic cups. **c** Electrostatic net (arrow) treated by *B. bassiana*, used for exposing adult *An. gambiae* to fungus spores. **d** Exposure cup with the *B. bassiana-*treated electrostatic net inside

This experiment consisted of four groups: one control and three treatment groups. In the control group, larvae were not exposed to predators. In the treatment groups, larvae were exposed to one, two or four *P. flavescens* nymphs. Each nymph was constrained in a separate clear, plastic cup with small holes (small enough to allow water circulation but no passage to the larvae), in the larval bowl with *An. gambiae* larvae (Fig. 1a, b). Each nymph was fed on 13 larvae per day. Each group was replicated four times with each replicate consisting of 30 first-instar (1-day-old) larvae placed in the larval bowl with 1 liter of water and observed till pupation day. Development time was recorded as number of days for the larva to develop into a pupa.

### The efficacy of *B. bassiana* against *An. gambiae* larvae

The efficacy of *B. bassiana* against *An. gambiae* larvae was determined by recording the number of dead mosquito larvae after fungal exposure. There were four groups: one control and three treatment groups. Each group had four replicates of 30 first-instar larvae in a bowl with 1 liter of water. The control group was not exposed to the fungus, but the treatment groups were exposed to 3 mg, 6 mg or 12 mg of fungal spores. Fungal spores were dusted on the water surface in the larval bowls. The number of dead larvae was recorded daily until all larvae had either died or pupated. The larvae were fed during the experiment with ground cat food (as mentioned above) and the amount of food was adjusted based on the daily mortality [23].

### Survival of adult mosquitoes exposed to fungus *B. bassiana* after predator and/or parasite larval pre-exposure

This experimental set up comprised of one control group and four treatment groups: Group I, no larval or adult exposure (control); group II, no larval exposure and adults exposed to fungus; Group III, larvae exposed to constrained predator and adults exposed to fungus; Group IV, larvae exposed to fungus and adults exposed to fungus; and Group V, larvae exposed to both predator and fungus and adults exposed to fungus.

### Larval infection

It was determined from the experiments of the second and third objectives that 2 dragonfly nymphs and 6 mg of fungus spores reduced the survival of the *An. gambiae* larvae but allowed some to pupate. Two nymphs were each constrained separately in a 300 ml clear plastic cup with small holes, which were placed in the same larval bowl that contained the larvae. These nymphs were fed on 13–14 mosquito larvae every day. Larvae were exposed to 6 mg fungus as described earlier. Dead larvae and pupae were separately recorded and removed daily in all the groups. To reach the minimum of 30 adult female mosquitoes per replicate for adult exposure to fungus, the number of larvae exposed to fungus was increased to adjust for the expected larval mortality in treatments. This was based on the results of a previous experiment. However, to ensure that there was no effect of density on larval development, the larvae were exposed to fungus in groups of 30 larvae per tray [23]. In total 15 trays were used to rearing the larvae.

The developing pupae were placed in 300 ml transparent plastic cups, which were covered with a mosquito net. Cotton wool soaked in 6% glucose solution was put on the net. The females that developed from these pupae were kept in holding cages according to their group.

### Adult infection

Only 2–3-day-old females were exposed to fungal infection. Each of the five groups had three replicates, with 30 females. In the treatment group the females were infected with *B. bassiana* fungus using electrostatic net (Fig. 1c). The net was cut into 22 × 11 cm size pieces and placed in plastic bowl that contained 5 g fungal spores. The bowl was covered with its lid, shaken thoroughly and allowed to rest for 5 min for the fungal spores to settle down. The net, now full of fungal spores was removed and fixed inside a 300 ml cup with staple pins to construct the exposure cup (Fig. 1d). The spore density on the electrostatic nets that the adult mosquitoes were exposed to was 3.1 × 10^8^ spores/242 cm^2^ (1.28 × 10^6^ spores/cm^2^). The method used to calculate spore density is described in Additional file 1: Text S1. One female mosquito, at a time, was aspirated from the holding cage and introduced into the exposure cup with the fungus-exposed electrostatic net (or for the control, only the electrostatic net) [27]. The top of the cup was covered with mosquito net and the female was left inside for 10 min. After that, the female mosquito was transferred into a holding cup containing 6% glucose solution-soaked cotton wool and monitored daily till death. The procedure was repeated for all the 360 female adult mosquitoes in the treatment groups.

Dead females were removed from their holding cups and confirmed for fungal infection. This was achieved by dipping cadavers in 70% ethanol to remove external microbiota (which does not affect the internally growing fungus) [29], then incubated on moist filter paper in sealed Petri dishes at 27 ± 1 °C. After 3–5 days, mosquito cadavers were examined for fungal sporulation, specifically emerging hyphae, using a dissecting microscope [25].

### Statistical analysis

To determine the predation efficacy of *P. flavescens* nymphs against *An. gambiae* larvae, the lethal median time to death (LT50) was calculated by Probit analysis of larval survival over time. Mann–Whitney U-test was used to determine the difference in the mean number of dead (missing) larvae in the control and treatment groups after 24 h. Kaplan–Meier (KM) pairwise comparison was used to determine the difference in development rate (from first-instar larvae to the pupal stage) among *An. gambiae* larvae reared in the presence of varying densities of *P. flavescens* nymphs. Cox regression was used to determine difference in the likelihood of pupation (the event) in the control and treatment groups in terms of hazard ratio (HR) [33]. The efficacy of varying dosage of *B. bassiana* against *An. gambiae* larvae was also determined by Cox regression. In the experiments on survival of adult mosquitoes exposed to the fungus after pre-exposure to predator and/or parasite, LT50 (95% CI) was calculated by KM analysis of adult survival over time and KM pairwise comparison was used to compare the groups. Cox regression was used to determine the difference in survival rates of adult mosquitoes in the control and treatment groups. To determine if there was synergy between predator and parasite exposure, mortality rates of the combination of predator and parasite (observed) were compared with the sum of mortalities induced by each, predator and parasite, separately (expected). The expected mortality was calculated using the formula Mexp = Mb + Mp (1 − Mb/100), where Mb and Mp are the observed percent mortalities caused by the parasite and the predator alone, respectively. The expected and observed mortalities were compared using a paired samples t-test [29]. SPSS version 17 was used for all statistical analyses.

## Results

### Predation efficacy of *P. flavescens* nymphs against *An. gambiae* larvae

**Fig. 2:**
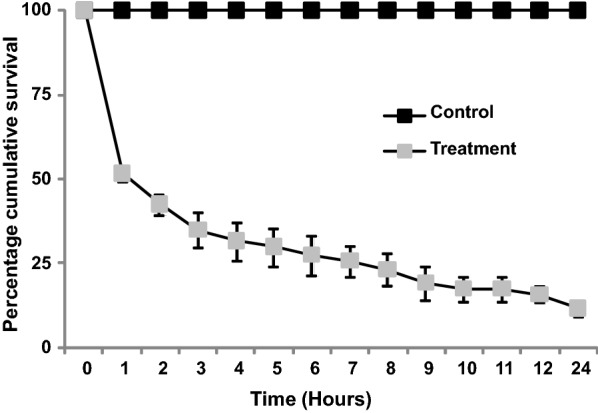
Percentage cumulative survival (± standard error, SE) of *Anophes gambiae* larvae over 24 hours. Control group larvae were not exposed to *Pantala flavescens* nymphs while the treatment larvae were exposed to one free *P. flavescens* nymph

No larval mortality was recorded in the control group within 24 h (no carcasses or injured larvae were found). In contrast, there was high larval mortality in the treatment group within the first hour (Fig. 2), with an average mortality of 88.3% (95% CI: 87.5–89.1%) after 24 h. In the treatment group, the LT50 calculated by Probit analysis (Slope ± SE = − 1.74 ± 0.48, *df *= 11, *P *< 0.001) was 0.6 (95% CI: 0.25–1.0) h. There was a significant difference in the mean number of missing larvae between the control and treatment groups at 24 h (Mann–Whitney U-test, *Z *= − 12.667, *U *= 1140, *n*_1 _= *n*_2 _= 120, *P *< 0.001) (Fig. 2).

### Development rate of *An. gambiae* larvae reared in the presence of varying densities of constrained *P. flavescens* nymph

**Fig. 3:**
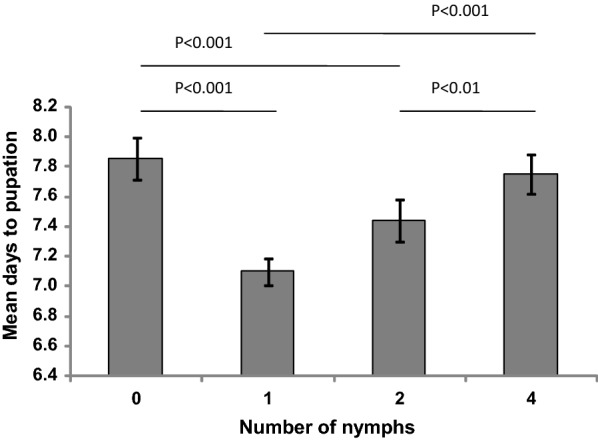
Mean days to pupation (± standard error, SE) of *Anopheles gambiae* larvae exposed to constrained 0, 1, 2 or 4 *Pantala flavescens* nymphs. The groups that are significantly different (Kaplan–Meier pairwise comparison) are indicated with *P*-values

Varying the density of the constrained predator had a variable effect on the development rates of *An. gambiae* larvae over time. Larval mortality was recorded as: 5% (95% CI: 4.5–5.5%) in the control group; 2.5% (95% CI: 2.1–2.9%) in the group exposed to one nymph; 9.2% (95% CI: 8.9–9.4%) in the group exposed to two nymphs; 5.8% (95% CI: 5.5–6.2%) in the group exposed to four nymphs. The surviving larvae in the control group took the longest period, a mean of 7.85 (95% CI: 7.8–8.0) days, to develop to pupae. When larvae were exposed to only one nymph, the mean number of days to pupation was 7.1 (95% CI: 7.0–7.2), which was the shortest development period across all the groups. Larvae exposed to two nymphs had a mean of 7.4 (95% CI: 7.3–7.5) days to pupation, while larvae exposed to four nymphs had a mean of 7.75 (95% CI: 7.6–7.9) days to pupation (Fig. 3). Kaplan–Meier pairwise comparison showed a significant difference in number of days to pupation between control group larvae and the larval groups exposed to one (*χ*^2 ^= 61.5, *P *< 0.001, *n *= 120) and two nymphs (*χ*^2 ^= 14.1, *P *< 0.001, *n *= 120), but no difference between the control group larvae and the larval group exposed to four nymphs (*χ*^2 ^= 1.5, *P *< 0.23, *n *= 120). The number of days to pupation in the larval group exposed to four nymphs was significantly different from that in the larval groups exposed to one (*χ*^2 ^= 54.6, *P *< 0.001, *n *= 120) and two nymphs (*χ*^2 ^= 8.2, *P *< 0.01, *n *= 120) (Fig. 3).

Hazard ratio (HR) values obtained from Cox regression analysis showed that, compared to the larvae in the control group, larvae exposed to one nymph were twice [HR = 2 (95% CI: 1.5–2.6); *P *< 0.0001, *df *= 1] as likely to pupate while larvae exposed to two nymphs were 1.4 times [HR = 1.4 (95% CI: 1.1–1.8); *P *< 0.02, *df *= 1] more likely to pupate (Table 1). However, larvae exposed to four nymphs were as likely to pupate as the control group [HR = 1.1 (95% CI: 0.8–1.4); *P *< 0.50, *df *= 1].

**Table 1:** Hazard ratios (HR) (event:pupation) of *An. gambiae* larvae exposed to varying numbers of constrained *P. flavescens* nymphs

No. of nymphs	HR	95% CI	*P*-value	*n*	*df*
0	1	–	–	120	3
1	2.0	1.5–2.6	**< 0.0001**	120	1
2	1.4	1.1–1.8	**0.02**	120	1
4	1.1	0.8–1.4	0.50	120	1

*Note*: Significant *P*-values (Cox regression) are shown in bold

*Abbreviations*: *n*, sample size; *df*, degrees of freedom

### Efficacy of varying dosage of *B. bassiana* against *An. gambiae* larvae

**Fig. 4:**
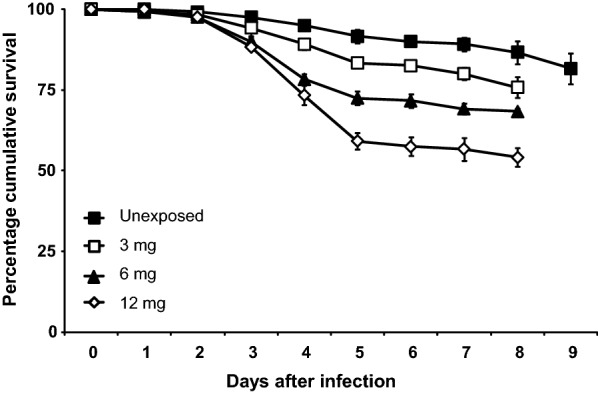
Percentage cumulative survival (± standard error, SE) of *Anopheles gambiae* larvae unexposed to *Beauveria bassiana* spores and exposed to 3, 6 and 12 mg of spores

The germination percentage of fungus spores used for this experiment was 97%. All treatment (fungus-exposed) groups had lower survival than the control group (un-exposed) and there was a clear dose-response (Fig. 4). Cox regression analysis showed a significant difference between the survival of larvae in the control group and all larvae in the treatment groups exposed to 3 mg, 6 mg and 12 mg of fungus. The analysis showed that, compared to the control group, larvae exposed to 3 mg of fungus were twice as likely to die [HR = 2.0 (95% CI: 1.2–3.3); *P *= 0.01, *df *= 1] than the control larvae, while those exposed to 6 mg and 12 mg of fungus were 2.5 [HR = 2.5 (95% CI: 1.5–4.2); *P *< 0.0001, *df *= 1] and 3.5 [HR = 3.5 (95% CI: 2.2–5.7); *P *< 0.0001, *df *= 1] times, respectively, more likely to die compared to the control larvae (Table 2).

**Table 2:** Hazard ratios (HR) of *An. gambiae* larvae exposed to varying doses of *B. bassiana* spores

Fungal dose (mg)	HR	95% CI	*P*-value	*n*	*df*
0	1	–	–	120	3
3	2.0	1.2–3.3	**0.01**	120	1
6	2.5	1.5–4.2	**< 0.0001**	120	1
12	3.5	2.2–5.7	**< 0.0001**	120	1

*Notes:* Significant *P*-values (Cox regression) are shown in bold

*Abbreviations*: *n*, sample size; *df*, degrees of freedom

### Survival of adult mosquitoes exposed to fungus *B. bassiana* after predator and/or parasite larval pre-exposure

Kaplan–Meier analysis of survival data on adult mosquito exposure to fungus following larval pre-exposure to predator and/or fungus indicate that control group adults had an LT50 of 23 days, which is longer than the LT50 of all treatment groups (Table 3). All mosquito cadavers in the treatment groups were positive for fungal infection (hyphae observed under the microscope), while mosquito cadavers from the control group were negative.

**Table 3:** Lethal median time (LT50) and hazard ratios (HR) of *An. gambiae* adults that emerged from unexposed larvae or larvae exposed to two *P. flavescens* nymphs and/or 6 mg *B. bassiana* fungus spores

Group	Larval exposure to		Adult exposure to fungus	LT50 (95% CI) (days)	Hazard ratio				
	Nymph	Fungus			HR	95% CI	*P*-value	*n*	*df*
I	No	No	No	23 (20–26)	–	–	–	90	4
II	No	No	Yes	5 (4.7–5.3)	45.8	17.8–117.7	**< 0.001**	90	1
III	Yes	No	Yes	4 (3.8–4.1)	67.4	26.0–174.2	**< 0.001**	90	1
IV	No	Yes	Yes	5 (4.7–5.3)	50.9	19.8–130.8	**< 0.001**	90	1
V	Yes	Yes	Yes	4 (3.7–4.3)	112.0	43.3–289.5	**< 0.001**	90	1

*Notes*: Significant *P*-values (Cox Regression) are shown in bold

*Abbreviations*: *n*, sample size; *df*, degrees of freedom

**Fig. 5:**
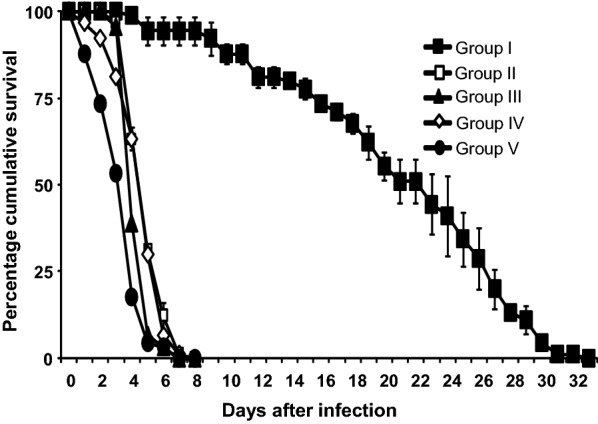
Percentage cumulative survival (± standard error, SE) of *Anopheles gambiae* adults. Group I, unexposed; Group II, exposed to *Beauveria bassiana* fungus at the adult stage; Group III, exposed to *Pantala flavescens* nymphs at the larval stage and *B. bassiana* at the adult stage; Group IV, exposed to *B. bassiana* at the larval and adult stage; Group V, exposed to both *P. flavescens* nymphs and *B. bassiana* at the larval stage and *B. bassiana* at the adult stage

Considering the lethal median time (LT50), control group adults (Group I) survived longer than adults not pre-exposed to any factor during the larval stage (Group II), adults pre-exposed to predator (Group III), adults pre-exposed to fungus (Group IV) and adults pre-exposed to both predator and fungus (Group V) (Fig. 5).

Hazard ratios indicated that adults in Group V were most likely to die after fungal exposure [HR = 112 (95% CI: 43.3–289.5); *P *< 0.001, *df *= 1] followed by Group III [HR = 67.4 (95% CI: 26–174.2); *P *< 0.001, *df *= 1)]. Kaplan–Meier pairwise comparison showed that exposure to fungus during the larval stage had no effect on the fungus susceptibility at the adult stage, as Groups IV and II were not significantly different (*χ*^2 ^= 1, *P *= 0.316) (Table 4). However, exposure to *P. flavescens* nymph during the larval stage increased the susceptibility to fungus at the adult stage as Groups III and II were significantly different (*χ*^2 ^= 16.7, *P *< 0.001) (Tables 3, 4). Exposure to only *P. flavescens* nymph or fungus spores during the larval stage influenced the susceptibility of adults to fungus spores, with mosquitoes exposed to the nymph during the larval stage being more susceptible. This was indicated by the significant difference between Groups III and IV (*χ*^2 ^= 8.67; *P *= 0.003) (Table 4) and Group III showed a higher hazard rate compared to Group IV [HR = 67.4 (95% CI: 26.0–174.2); *P *< 0.001, *df *= 1 *versus* HR = 50.9 (95% CI: 19.8–130.8); *P *< 0.001. *df *= 1] (Table 3). The synergistic effects of predation stress and fungal infection on adult longevity (unexposed to fungal infection) were not significant (*P *= 0.22), despite a mean difference of 6% in the observed and expected mortality.

**Table 4:** Kaplan–Meier pairwise (*P-*values) comparison of *An. gambiae* groups

Group	I		II		III		IV	
	*χ*^2^	*P*-value	*χ*^2^	*P*-value	*χ*^2^	*P*-value	*χ*^2^	*P*-value
II	178.6	< 0.001						
III	182.0	< 0.001	16.7	< 0.001				
IV	174.8	< 0.001	1.00	**0.316**	8.67	0.003		
V	187.6	< 0.001	50.9	< 0.001	24.0	< 0.001	34.8	< 0.001

*Notes*: *Anopheles gambiae* groups: I, unexposed; II, exposed to *Beauveria bassiana* fungus at the adult stage; III, exposed to *P. flavescens* nymphs at the larval stage and *B. bassiana* at the adult stage; IV, exposed to *B. bassiana* at the larval and adult stage; V, exposed to both *P. flavescens* nymphs and *B. bassiana* at the larval stage and *B. bassiana* at the adult stage. Non-significant *P*-values (Kaplan–Meier pair wise comparison) are shown in bold

## Discussion

The results of this study confirmed the predation efficacy of *P. flavescens* nymphs and virulence of *B. bassiana* spores against *An. gambiae* larvae. It also confirmed the non-lethal effects of constrained *P. flavescens* nymphs on the development and survival of *An. gambiae* larvae. Interestingly, the study showed that larval exposure to *P. flavescens* nymphs increased the susceptibility of adults to *B. bassiana* spores, but larval exposure to *B. bassiana* spores did not influence the adult susceptibility to *B. bassiana* spores.

Nymphs of *Pantela flavescens* have a high predator efficacy against *An. gambiae* larvae feeding on almost 26 third-instar larvae within 24 hours. High predation efficacy of *P. flavescens* and other species of dragonfly nymphs against *An. arabiensis* and *Aedes aegypti* larvae has been reported [20, 21]. Similarly, high predation efficacy was also reported by Ameka [34], in which third-instar larvae of *An. gambiae* were exposed to nymphs of an unspecified species of dragonfly to determine their predation efficiency. Despite the high predation efficacy reported in the present study, it is important to note that the study set-up was experimental; therefore, it is not clear whether similar predation levels occur in the natural breeding sites and in presence of alternative prey.

Our results show that the effect on the development rate of mosquito larvae under predation risk was dependent on the density of the predator. The development time of *An. gambiae* larvae reared in the presence of one or two constrained *P. flavescens* nymphs was shorter than that for larvae reared in the absence of *P. flavescens* nymphs. However, the development time of *An. gambiae* larvae reared in the presence of four constrained *P. flavescens* nymphs and those reared in the absence of *P. flavescens* nymphs was the same. The reduced development time is in contrast to studies such as Stoks et al. [12] and Beketov et al. [35] showing an increase in the development time of prey under predation risk, but consistent with Culler et al. [37] and Zuhara et al. [36]. In general, threat-sensitive response hypothesis predicts that prey adjust the time spent on antipredator response according to the threat level to allow as much time as possible for foraging and development [38, 39]. However, the effect of predator on prey development is varied and depends on both the species of prey and predator, as well as on the hunting mode of the predator [39–41]. In the study conducted by Stoks et al. [12] the larvae of damselfly *Lestes viridis* were exposed to one confined stickleback predator fish *Gasterosteus aculeatus*. As a result, the prey was exposed to both visual cues and predator kairomones. The stickleback fish has a similar hunting mode to dragonfly nymphs, where it sits, pursues, attacks and captures its prey. However, damselfly larvae are cannibalistic, and the conspecific cues might have also played a role, resulting in slower development time. Beketov et al. [35], unlike in this study, exposed the *Culex pipiens* mosquito larvae only to the cues of the predator *Notonecta glauca* that had been fed on *Culex* larvae or another prey like *Daphia magna*. The feeding could have resulted in addition of alarm pheromones, produced by the prey before being predated on by *N. glauca*, into the predator cues of *N. glauca*. This might have resulted in the delayed development of the *Culex* larvae. It is also known that predator cues produced by a predator that hunts in a sit and pursue mode, like dragonfly nymphs, have a stronger non-lethal effect than a predator that hunts actively like *N. glauca* [40]. In the study by Culler et al. [37], the predator dytiscid beetle larvae use a variety of hunting modes, including sit-and-wait and active hunting, the former being similar to the dragonfly nymphs [40, 42].

As the number of *P. flavescens* nymphs increased in the bioassays, the development time of the *An. gambiae* larvae increased so that it was equal to the unexposed larvae in the presence of four nymphs. There can be three explanations for this: (i) cannibalism among the predator; (ii) sensory habituation; and (iii) risk assessment by the prey [43]. The first explanation is cannibalism among the predator. Studies mentioned above were carried out with either one predator in the bioassay or predator cues [12, 35, 37]. Aquatic predators, i.e. backswimmers, diving beetles and dragonfly nymphs are known to be cannibals [44–46]. Dragonfly nymphs have a density-dependent cannibalism and both visual and chemical cues play a role in determining the presence of a conspecific nymph [46, 47]. These cues reduce the foraging activity of the dragonfly nymphs [46]. As the nymph density increased, the chemical cues may have changed releasing the mosquito larvae from the effects of intimidation by the predator [48]. However, in the present study, nymphs of the same weight were used and chemical cues from larger conspecifics has a stronger effect on smaller individuals [46]. The second explanation is the sensory habituation of the larvae to the high concentration of predator cues due to the presence of four nymphs. The high concentration is indicative of imminent attack, which was never experienced due to the fact that the *P. flavescens* nymphs were confined. As a result, the larvae developed sensory habituation that resulted in either a lower anti-predator behavior or the receptors for the specific predator cues stopped responding due to continuous stimulation [43]. The latter is similar to the principle behind “mating disruption” used for pest control in agriculture [49]. The third explanation is risk assessment. In the present experiments, the larvae under low predation risk (1 predator) increased their foraging activity to escape the risky larval site, as soon as possible. However, under high predation risk (4 predators), the foraging activity was assessed by the larvae as high risk. Consequently, the larvae adopted a more basal foraging rhythm to be less conspicuous [38, 43, 50].

*Beauveria bassiana* fungus negatively affected the survival of *An. gambiae* larvae and the effect was dose dependent. The control larvae which were not exposed to the fungal parasite survived longer than the treatment larvae. This is consistent with the studies of Bukhari et al. [23] and Vogels et al. [18]. *Anopheles* larvae hang horizontally to the water surface because of their short siphons and are surface feeders. The spores kill the larvae by either contact with the epicuticle or by being ingested together with food, leading to infection. Spores that enter the larval body through the mouth or siphon also mechanically block these passages [23]. The attached spores germinate, releasing endotoxins as well as damaging the larval tissues during their vegetative growth [51]. These varied modes of action of the fungus lower the probability that resistance will develop against the fungus [23]. The presence of more spores on the water surface increases the likelihood of the larvae coming in contact with the spores and feeding, hence, the observed dose-dependent effect [14, 22, 23]. However, at higher doses, the fungus spores tend to clump together due to their hydrophobic nature and the dose-dependent effect is either not observed or is not proportional [23].

The survival of adult mosquitoes exposed to *B. bassiana* fungus after larval pre-exposure to the predator and/or parasite showed that predator presence in the larval breeding sites can influence the susceptibility to parasites in the adult stage. To our knowledge, this is the first study that indicates the influence of predator presence during the larval stage, on the susceptibility to a fungal entomopathogenic fungus parasite at the adult stage of *An. gambiae* mosquitoes. A study carried out by Yin et al. [52] showed that *Daphnia* water fleas exposed to fish kairomones were more susceptible to the yeast parasite *Metschnikowia*. In another study, it was found that above ground predators attacked the juvenile stages of a herbivore beetle *Leptinotarsa decemlineata* feeding on plant foliage, and made them more susceptible to the below-ground parasites that attacked the pupal stage resulting in high mortality of the beetle [53]. A possible explanation is the trade-off between anti-parasite and anti-predator defenses [16]. Exposure to predators is known to reduce the ability of hosts to cope with parasitism mediated through effects on immune function [54]. Perceived risks of predation and parasitism alters the components of the immune system such as haemocyte density and phenoloxidase activity [55]. However, the results of our study is in contrast to the study of Roux et al. [16], which showed that exposure to the predator backswimmer (*Anisops jaczewskii*) did not significantly alter mosquito (*An. coluzzii*) susceptibility to *P. falciparum*. One explanation can be the active hunting mode of *A. jaczewskii* that led to lower non-lethal effects on the host by reducing the adult size, fecundity and longevity [16, 40]. Another explanation may be that, in the study of Roux et al. [16], the predator was free to prey upon mosquito larvae which could have suppressed the weakest larvae and selected the strongest. The strong larvae were consequently able to resist several stresses. In our study, body size, survival and fitness of the adult *An. gambiae* mosquitoes were not considered. Another possible explanation may also be the mosquito innate immunity which possesses a fine-grained capacity to distinguish between classes of closely related eukaryotic parasites, hence, the difference observed in the susceptibility of the adults (pre-exposed to predator during the larval stage) after exposure to *B. bassiana* and *P. falciparum* [56, 57].

Parasite presence in the breeding site did not influence the susceptibility to parasite at the adult stage. This result is consistent with Vogels et al. [18] that showed that there was no significant difference in survival of *A. stephensi* females that were only exposed during the adult stage and females that were exposed during both the larval and adult stage. The study by Vogels et al. [18] also showed that only 14% of the females carried the infection from the larval stage. The similar susceptibility of mosquito adults to parasite in both groups (exposed or unexposed to parasite at the larval stage), is therefore more likely to be due to the low percentage of individuals carrying the infection and not due to an immune response. Indeed, it is noteworthy that the longevity of females that carried the infection from the larval stage was reduced compared to females that were not exposed during the larval stage. The longevity of females that did not carry infection from the larval stage was similar to females that were not exposed during the larval stage. In our study, the same individuals were used for larval and adult exposure, so we were unable to confirm if the infection was carried from the larval stage or was the result of adult exposure. Vogels et al. [18] considered this result an advantage, as control of both larval and adult stages using entomopathogenic fungi can complement each other. As far as the susceptibility to fungus is concerned, the results of our study showed that the presence of both predator and parasite have an additive effect.

The carry-over effects of dragonfly nymph, i.e. increased susceptibility to the fungus, can have an indirect effect on malaria transmission. There are conflicting studies regarding the reduced potential of malaria transmission by *Anopheles* mosquitoes when co-infected with fungus and *Plasmodium* during the adult stage [58, 59]. However, if indeed co-infection with fungus can reduce the potential of adult *Anopheles* mosquito to transmit malaria, the predator presence during the larval stage may enhance the reduction in malaria transmission [17]. It would be interesting to know if the predators can also increase the susceptibility to transgenic fungi that are actually designed to kill human malaria parasites [60].

Nonetheless, increased susceptibility to *B. bassiana* can influence malaria transmission by reducing the survival and influencing the behavior of the infected adult mosquitoes [61]. Also, predator presence in breeding sites can complement control programmes that deploy entomopathogenic fungi [30, 62]. This and follow-up studies may be used for informed decision-making on whether a mosquito breeding site needs to be treated or better left untreated when targeting the larval stage. Predators are generally present in large and semi-permanent or permanent breeding sites, e.g. rice paddies. Although easy to locate, they have a large surface area that needs to be treated, which is mired by the vegetation as well as the economic cost [63]. Large breeding sites that harbor predators, like dragonfly nymphs, would therefore be better left untreated to complement a control programme based on entomopathogenic fungi. However, further studies are needed to confirm this.

There are two main points to consider when interpreting the results of our study. First, the experiments were carried out in a laboratory setting with reared *An. gambiae*. Secondly, the predator was constrained. A free swimming and feeding predator may not have the same effect. Whether or not the predator presence can increase the susceptibility of *Anopheles* adults under natural conditions needs to be investigated.

## Conclusions

Both the parasite *B. bassiana* and the predator *P. flavescens* reduced the survival of *An. gambiae* larvae. Predator presence can also influence the development time of larvae. The surviving adults showed no increase in susceptibility to *B. bassiana* when pre-exposed to the same parasite during larval stage, but an increased susceptibility to *B. bassiana* was observed when adults were pre-exposed to the predator *P. flavescens* during the larval stage. This indicates that in addition to reducing the survival of malaria mosquitoes in the breeding site, the predator may also impact the immune response of the surviving individuals, making them less suitable for disease transmission. However, studies need to be carried out in field conditions for a realistic perspective.

## Supplementary information

**Additional file 1.** Calculation of fungus spore density on electrostatic net.

## Data Availability

Data supporting the conclusions of this article are provided within the article. The datasets used and/or analysed during the present study are available from the corresponding author on reasonable request.

## Abbreviations

WHO: World Health Organization
IVM: Integrated vector management
HR: Hazard ratio
