# Supplementary material for: Direct and indirect effects of predation and parasitism on the Anopheles gambiae mosquito
Source: Parasit Vectors. 2020 Jan 30;13:43. doi: 10.1186/s13071-020-3915-8 (PMC6990496; doi:10.1186/s13071-020-3915-8)
Supplement: Supplementary file 1 — Additional file 1. Calculation of fungus spore density on electrostatic net. [file 13071_2020_3915_MOESM1_ESM.docx]

**Additional file 1: Text S1. Calculation of fungus spore density on electrostatic net**

Adult *Anopheles gambiae* were exposed to an electrostatic net to infect them with fungus spores. Electrostatic net was impregnated with fungus spores. The fungus spore density on the electrostatic net was calculated as follows.

Spore count was done by cutting a 1 cm^2^ piece of the electrostatic net, which was placed in the bowl with 5 g fungus then shaken and left for 5 minutes to settle.

Thereafter, 0.1% Tween 80 solution was prepared, and 8 ml placed in 10 ml vial, which was replicated three times (Tween 80 solution suspends the fungal spores).

After 5 minutes, when the fungus in the bowl had settled, the net was picked by a pair of forceps and placed in the vial then shaken rapidly to dislodge the fungal spores from the electrostatic netting into the 1% Tween 80.

Then, 0.1µl of the solution with suspended fungal spores was taken using a micro-pipette and administered on a haemocytometer then covered with a cover slip.

The number of spores lying within the central grid of the haemocytometer were counted at x100 magnification under a compound microscope and recorded. This was repeated five times in different zones and the average calculated.

The average was used to estimate the number of spores in the 8 ml vials and therefore, on the 1 cm^2^ piece of the electrostatic net.
